# The Nature and Treatment of Deafness, Etc.

**Published:** 1849-07

**Authors:** 


*The Nature and treatment of Deafness and diseases of the ear; and the treatment of the Deaf and Dumb.* By WM. DUFTON, M. R. C. S., Philadelphia. Lea & Blanchard. 1848. 18 mo. pp., 120.

The treatment of diseases of the ear, as the author remarks in his preface, is not only much neglected by the profession, but has been, to a great extent, consigned to ignorant pretenders. The present work has been prepared with the hope of calling the attention of the profession and public to the important class of diseases of which it treats, and with a view to rescue their treatment from the hands of Empirics. The objects are certainly worthy, and, as the work is small, the reader can, with trifling expense and trouble, satisfy himself with respect to their fulfilment.

---

*Local Treatment of Dysentery.*—Having employed, recently, in a case of severe and protracted dysentery, with much satisfaction, some local remedies which, so far as our knowledge extends, are not in common use, we suggest them for consideration and trial by our professional brethren.

The parts inflamed in this disease are the colon and rectum, but more particularly, the latter. The anatomical characters, in fatal cases, are generally greatest at the lower part of the large intestine. All practitioners are familiar with the fact that the tenesmus incident to the affection, is the chief source of suffering. To relieve this distressing symptom, enemas containing opium, or morphia, are eminently serviceable, provided they are retained sufficiently for the anodyne effect to be induced; but we are often deprived of this valuable means of comfort, in consequence of the extreme irritability of parts occasioning the immediate expulsion of the injected fluid.

In tedious cases, it is generally the case that the intestine loses, after a time, the power of retention, so that this mode of administering anodyne remedies ceases to be available. In view of the circumstances just stated, it must be evident that remedies locally applied ,by enema, which are calculated to exert a salutary action over the portion of mucous membrane most highly inflamed, and to relieve the irritability of the rectum, so that suffering from tenesmus is diminished, and the power of retention increased, cannot but exert a salutary influence in the treatment of this painful and severe affection. The nitrate of silver, as we know, in analogous instances of inflamed mucous tissue, for example, in conjunctivitis, pharyngitis, etc., exerts a surprising effect in diminishing and arresting inflammatory action. It has been employed, to some extent, in dysentery, and is recommended by some practical writers, but, so far as we know, is by no means in common use. In the case referred to, we resorted to a solution of the crystals of the nitrate of silver, ten grains to the ounce, with marked benefit. The tenesmus and frequent dejections were relieved in a striking degree, and the discharge of mucus and blood was much diminished. To secure the good effects of this application, it is desirable that the injection be made to pass up the intestine as high as practicable, in order to bring it into contact with a larger portion of the inflamed surface. We found the best instrument at hand, to be a female bone syringe, with a long pipe, terminating by a perforated bulbous extremity. Perhaps a solution of greater strength might be even more serviceable. The patient was a child four years of age. The application occasioned, apparently, little or no pain—not more than the ordinary enemas of starch and laudanum.

Another remedy, employed in the same case, was a creasote mixture. For the suggestion of this remedy, we are indebted to our friend and former pupil, Dr. Rogers, of Spencerport, Monroe county, N. Y. Dr Rogers informed me that he had employed creasote injections in several cases of severe dysentery, with very excellent results, in one case, which had resisted other resources, the relief being immediate and almost complete—the remedy acting, in fact, like a charm. He thinks the life of the patient in that case, was saved by resorting to it. He derived the suggestion from an article contained in one of the early numbers of Braithwaite's Retrospect. We have used this remedy in two cases; in one of chronic dysentery of long standing, the effect was good, but not extraordinary. In the case recently under treatment, we first employed it in connection with the tincture of opium, and found that the enemas were retained, when, with the laudanum alone, they were immediately expelled. We employed, at first, a mixture for each injection (℥ss) containing two minims of creasote. Subsequently we employed the creasote alone, increasing the quantity to four minims, and the good effects were striking. The relief of the local symptoms was quite as great as when the opium was given in combination, the disadvantages of the latter being avoided. We feel confident that this will prove a valuable remedy in the treatment of dysentery, and we, therefore, are solicitous that our readers should make trial of it. Its salutary operation in some cases of gastric irritability is well known to many practitioners, and we might, from analogy, suspect that it prove equally efficacious in controlling a similar morbid condition in the other extremity of the alimentary canal. We do not, of course, suggest these as remedies intended to supersede other therapeutical measures, but only as useful auxiliaries thereto.

